# Inflammation on the Cervical Papanicolaou Smear: Evidence for Infection in Asymptomatic Women?

**DOI:** 10.1155/2013/184302

**Published:** 2013-09-24

**Authors:** Stavroula Baka, Ioanna Tsirmpa, Anthia Chasiakou, Iliana Tsouma, Ekaterina Politi, Vassiliki Gennimata, Evangelia Kouskouni

**Affiliations:** ^1^Biopathology Laboratory, University of Athens, Aretaieio University Hospital, 11528 Athens, Greece; ^2^Cytology Laboratory, University of Athens, Aretaieio University Hospital, 11528 Athens, Greece

## Abstract

*Background*. The significance of the possible presence of infection on the Pap smear of asymptomatic women based on cytological criteria is practically unknown. *Materials and Methods*. A total of 1117 asymptomatic nonpregnant women had Pap smear tests and vaginal as well as cervical cultures completed (622 with and 495 without inflammation on the Pap smear). *Results*. Out of the 622 women with inflammation on Pap test, 251 (40.4%) had negative cultures (normal flora present), while 371 (59.6%) women had positive cultures with different pathogens. In contrast, the group of women without inflammation on Pap test displayed significantly increased percentage of negative cultures (67.1%, *P* < 0.001) and decreased percentage of positive cultures (32.9%, *P* < 0.001). Bacterial vaginosis was diagnosed more frequently in both groups and significantly more in the group with inflammation on Pap smear compared to the group without inflammation (*P* < 0.02). *Conclusions*. A report of inflammatory changes on the cervical Pap smear cannot be used to reliably predict the presence of a genital tract infection, especially in asymptomatic women. Nevertheless, the isolation of different pathogens in about 60% of the women with inflammation on the Pap smear cannot be overlooked and must be regarded with concern.

## 1. Introduction

The infections of the genital tract are common in reproductive-age women and the associated cost is substantial. It has been noted that many women remain asymptomatic in the presence of vaginitis or cervicitis [1, 2]. The Papanicolaou (Pap) test is a simple, quick, and painless procedure performed on cells from the uterine cervix and used as a screening test for the prevention of the cancer of uterine cervix [3, 4]. When reporting the results of cervical Pap smears tests usually a remark is made on the possible presence of infection based on cytological criteria [5]. The clinical significance of inflammation on the Pap smear of asymptomatic women is practically unknown since no guidelines exist on appropriate management. Thus clinicians are often faced with the dilemma on whether these women should be counselled to present for vaginal/cervical cultures in order to isolate possible pathogens [6, 7]. This study assessed the possible association between inflammation on Pap smears and the presence of cervical/vaginal pathogens as determined by cultures.

## 2. Materials and Methods

The population studied consisted of asymptomatic nonpregnant women of reproductive age presenting to the Obstetrics and Gynaecology Outpatient Clinic of Aretaieio University Hospital for a routine Pap smear test between January 2008 and May 2012 and who gave consent to be included in this study. The Ethics Committee of Aretaieio Hospital approved the study, and informed consent forms were obtained from all subjects. 

Women were counselled to present to the hospital 10 to 20 days after the first day of their menstrual cycle without using vaginal douche or medication for at least a month. The smears showing inflammatory changes or no inflammation were included in this study, while inadequate smears and smears showing any atypical morphology were excluded. Inflammatory changes as detected on a Pap stained smear included the presence of cells with enlarged nuclei, pyknosis or karyorrhexis, perinuclear halos, and vacuoles. Often increased numbers of polymorphonuclear leukocytes or neutrophils and parabasal cells with generalised eosinophilia of the cells were recorded. Furthermore, the presence of epithelial cells covered with blue-stained coccoid bacteria on the stained cervical smear together with a decreased number or a lack of lactobacilli represented findings suggestive of bacterial vaginosis.

Asymptomatic women with or without inflammatory changes on their Pap smear were recalled for cultures. Genital tract samples (vaginal and cervical) were available for analysis. A wet mount as well as a gram-stained smear was examined under microscope to screen for *Trichomonas vaginalis* and to obtain valuable information about the microorganisms (yeasts and bacteria) present as well as for the diagnosis of bacterial vaginosis using both Amsel and Nugent criteria [8, 9]. The women were tested for the presence of different aerobic bacteria, that is, *Candida* species, group B streptococcus, *Gardnerella vaginalis*, and *Neisseria gonorrhoeae*, and anaerobic bacteria. Thus, clinical specimens collected from patients were inoculated onto appropriate plates for standard aerobic and anaerobic cultures and incubated at 37°C for 24 h and 48 h, respectively. The isolated pathogens were identified using the automated system VITEK 2 (BioMerieux, Marcy l'Etoile, France). Furthermore, the presence of *Chlamydia trachomatis* as well as *Ureaplasma urealyticum* and *Mycoplasma hominis*, in the specimens studied, was determined using the COBAS AMPLICOR *Chlamydia trachomatis* test (Roche Diagnostics, USA) and *Mycoplasma* IST2 (BioMerieux, Marcy l'Etoile, France), respectively.

Statistical analysis was performed using chi square test, and values ≤0.05 were considered as significant.

## 3. Results

A total of 1117 women had smear tests and vaginal as well as cervical cultures completed (622 with and 495 without inflammation on the Pap smear). Culture results in the population studied are presented in Table 1. Women with inflammation on Pap test displayed significantly decreased percentage of negative cultures (*P* < 0.001) and increased percentage of positive cultures (*P* < 0.001). Among the women with genital tract pathogens detected, bacterial vaginosis was diagnosed more frequently in both groups. Interestingly, in the group with inflammation on Pap smear, the women diagnosed with bacterial vaginosis were significantly more compared to the group without inflammation (*P* < 0.02). The isolation of other pathogens such as *Candida* species, *Chlamydia trachomatis*, and *T. vaginalis* and the cases diagnosed as aerobic vaginitis (characterized by the isolation of *Streptococcus agalactiae*, gram-negative rods, and methicillin-resistant *Staphylococcus aureus*) did not differ between the two groups.

## 4. Discussion

In our study we carried out vaginal and cervical cultures for a wide range of microorganisms in women with and without inflammation on Pap test in order to determine the predictive value of this finding for the presence of pathogens in asymptomatic women. We established that almost 60% of the women presenting with inflammatory changes on cervical smear tests had positive cultures for different pathogens. Cervicovaginal microbial flora characterization is necessary, and it would, thus, be opportune for all patients who present with an inflammation on Pap test to undergo a cervicovaginal microbiological examination to detect potential pathogens for correct diagnosis and treatment. Kelly and Black found that 47% of women with inflammatory changes on cervical smear testing had a microbiologically proven infection [10]. Wilson et al. also reported that inflammation on cytology is often associated with a genital tract infection [11]. Similar findings and comparable to our results were reported in the literature [2, 12, 13]. However, approximately one-third of the women with no inflammatory changes on smear testing had positive cultures. Previous studies demonstrated that women with no inflammatory changes on cervical smears can harbour genital tract pathogens. Bertolino et al. noted that inflammation on Pap smear had a relatively low predictive value for the presence of vaginal pathogens in asymptomatic women [5] while Parsons et al. found a high rate of positive cultures both in women with inflammatory changes and in those without inflammatory changes on smear testing, suggesting that inflammation on Pap test is a poor indicator of cervical infection [6]. On the other hand, Burke and Hickey demonstrated that the prevalence of infection was higher in the inflammatory smear group, thus supporting that women with an inflammatory smear are more likely to harbour genital tract infection than women whose smear shows no evidence of inflammation [7].

Bacterial vaginosis is a condition where an impressive microecologic alteration of vaginal flora takes place characterized by decreased *Lactobacillus* spp. and overgrowth of *Gardnerella vaginalis* together with anaerobes and potentially pathogenic bacteria, including *Ureaplasma urealyticum*, and *Mycoplasma hominis*. Inflammation on Pap smear has been associated with a 30–50% incidence of bacterial vaginosis [14, 15] which is consistent with our findings. Furthermore, others report that most of the smears with inflammatory changes contained clue cells, thus indicating bacterial vaginosis and concluding that Pap smear is well suited in diagnosing cervical infections associated with bacterial vaginosis [2, 7, 12].

The vaginal flora is a complicated microenvironment which consists of different bacterial species in continuously variable quantities and proportions. Although some pathologic conditions and clinical entities are well defined, the presence of some aerobic microorganisms can be regarded as colonization. In 2002, Donders et al. proposed the term aerobic vaginitis in order to better define a pathologic condition which is correlated with the presence of aerobic bacteria, mainly group B streptococci, *Escherichia coli*, and *Staphylococcus aureus* [16]. Interestingly, it has already been reported that *Streptococcus agalactiae* inhibits the growth of lactobacilli and *Gardnerella vaginalis*, but not *S. aureus* [17, 18]. In our study population, the prevalence of *S. agalactiae* and gram-negative rods (mainly *E. coli*) was lower than a recent report [19], but was similar to that reported by Donders et al. [16] and Lukic et al. [20].

*Staphylococcus aureus* vaginal colonization has been widely studied since toxic shock syndrome emerged some decades ago. However, vaginal carriage of methicillin-resistant *S. aureus* (MRSA) has not been well documented. The prevalence of MRSA was low in our population and similar to previous reports in ambulatory, nonhospitalized, and asymptomatic sample [16, 21].

Forty-three women (11.6%) with inflammation on Pap smear were positive for *Candida* infection with the majority of them being diabetic. These findings are consistent with previous results presented in the literature [16], yet not consistent with a recent report where, in a different population, the rate of *Candida* infection in patients with inflammatory changes was very high, 73.8% [13]. On the other hand, our findings are high compared to other reports in the literature [2, 6, 22].

Some studies report on the correlation between evidence of inflammation on smear testing and positive specimens for *Chlamydia trachomatis* [5, 7, 12]. In our sample, the prevalence of *C. trachomatis* positive testing was 0.8%, which is in accordance with previous reports [6, 13, 15]. Screening for lower genital tract infection, using either an intracervical or a vaginal swab, for *Chlamydia* infection should be performed even if in some populations the reported prevalence is low since Chlamydia infection, in particular, has long-term fertility consequences through its potential to cause asymptomatic tubal damage.

*Trichomonas vaginalis* is a parasite that causes symptomatic and asymptomatic infection of the female urogenital system. It has been proven that recovery of *T. vaginalis* was more frequent in women with inflammation on Pap smear than in women without inflammation [5, 12]. In our study, only two samples were positive for *T. vaginalis* in women with inflammation, which is consistent with others reporting low prevalence [16, 22, 23] and different from studies that found an increased frequency of this parasite [2, 13].

## 5. Conclusions

The results of our study suggest that a report of inflammatory changes on the cervical Pap smear cannot be used to reliably predict the presence of a genital tract infection, especially in asymptomatic women. Further studies with a longitudinal character are needed to look into the possible presence of infection based on cytological criteria. Nevertheless, the isolation of different pathogens in about 60% of the women with inflammatory changes on the cervical Pap smear, in the absence of *Lactobacillus* species, cannot be overlooked and must be regarded with concern.

## Figures and Tables

**Table 1:** Culture results in study groups. Figures are numbers (percentages).

Culture results	Inflammation (*n* = 622)	No inflammation (*n* = 495)	*P* value
Negative cultures	251 (40.4)	332 (67.1)	<0.001
Positive cultures	371 (59.6)	163 (32.9)	<0.001
Bacterial vaginosis	274	136	<0.02
*Candida* spp.	43	12	NS
* C. trachomatis *	3	0	NS
* T. vaginalis *	2	0	NS
Aerobic vaginitis	49	15	NS
